# Ischemic Burden and Clinical Outcome: Is One ‘Culprit’ Ischemic Segment by Dobutamine Stress Magnetic Resonance Predictive?

**DOI:** 10.1371/journal.pone.0115182

**Published:** 2014-12-17

**Authors:** Sorin Giusca, Sebastian Kelle, Eike Nagel, Sebastian Johannes Buss, Valentina Puntmann, Ernst Wellnhofer, Eckart Fleck, Hugo Albert Katus, Grigorios Korosoglou

**Affiliations:** 1 University of Heidelberg, Department of Cardiology, Heidelberg, Germany; 2 German Heart Institute Berlin, Department of Internal Medicine/Cardiology, Berlin, Germany; 3 King's College London, Division of Imaging Sciences, London, United Kingdom; Hospital de Clínicas de Porto Alegre, Brazil

## Abstract

**Aims:**

We sought to evaluate the impact of ischemic burden for the prediction of hard cardiac events (cardiac death or nonfatal myocardial infarction) in patients with known or suspected CAD who undergo dobutamine stress cardiac magnetic resonance imaging (DCMR)

**Methods:**

We included 3166 patients (pts.), mean age 63±12 years, 27% female, who underwent DCMR in 3 tertiary cardiac centres (University Hospital Heildelberg, German Heart Institute and Kings College London). Pts. were separated in groups based on the number of ischemic segments by wall motion abnormalities (WMA) as follows: 1. no ischemic segment, 2. one ischemic segment, 3. two ischemic segments and 4. ≥three ischemic segments. Cardiac death and nonfatal myocardial infarction were registered as hard cardiac events. Pts. with an “early” revascularization procedure (in the first three months after DCMR) were not included in the final survival analysis.

**Results:**

Pts. were followed for a median of 3.1 years (iqr 2–4.5 years). 187 (5.9%) pts. experienced hard cardiac events. 2349 (74.2%) had no inducible ischemia, 189 (6%) had ischemia in 1 segment, 292 (9.2%) in 2 segments and 336 (10.6%) ≥3 segments. Patients with only 1 ischemic segment showed a high rate of hard cardiac events of ∼6% annually, which was 10-fold higher compared to those without ischemia (0.6% annually, p<0.001) but similar to those with 2 and ≥3ischemic segments (∼5.5% and ∼7%, p = NS).

**Conclusions:**

The presence of inducible ischemia even in a single ‘culprit’ myocardial segment during DCMR is enough to predict hard cardiac events in patients with known or suspected CAD.

## Introduction

Estimating the risk of subsequent cardiac events is of paramount importance in patients with known or suspected coronary artery disease (CAD), because an invasive therapy is warranted for patients with myocardial ischemia who are at high-risk for future events [1].

Stress induced myocardial ischemia exhibits significant prognostic information in this population cohort, surpassing the prognostic value of conventional atherogenic risk factors [2]. Although similar sensitivity and specificity were reported in detecting ischemia induced wall motion abnormalities (WMA) for dobutamine stress echocardiography (DSE), nuclear perfusion imaging and high-dose dobutamine stress cardiac magnetic resonance (DCMR) [3], [4], [5], the latter offers the advantage of excellent spatial and temporal resolution and can be performed without ionizing radiation and even without the need for contrast agent administration for the patients.

The extent of ischemia was shown to carry prognostic value in patients undergoing DSE. In this regard, patients with ≥3 segments with WMA exhibit high-risk for subsequent cardiac events [6]. However, although current guidelines translate these findings to other imaging modalities, until now no data are available related to the prognostic value of ischemia extension for DCMR [7].

In this respect, the aim of our study was to determine whether (i) the extent and (ii) the localization of inducible myocardial ischemia in terms of WMA during high-dose dobutamine stress cardiac magnetic resonance (DCMR) are decisive for the prediction of cardiac events and future revascularization procedures.

## Methods

### Study population

The study was conducted in accordance with the standards of our local ethics committee (University Hospital Heidelberg, German Heart Institute, King's College London). Our institutional review board (University of Heidelberg, Alte Glockengießerei 11/1, D-69115 Heidelberg, http://www.medizinische-fakultaet-hd.uni-heidelberg.de/Ethikkommission.106025.0.html) specifically approved our study (Ethics committee registration number S-281/2008, clinical trial number NCT00837005). Written informed consent was obtained from all patients before the DCMR examination.

3166 Patients (63±12 years, 27% female) enrolled in 3 tertiary cardiac centres (University Hospital Heidelberg, German Heart Institute, Berlin and King's College, London) were evaluated using DCMR between January 2000 and June 2008. Examinations were performed using 1.5-Tesla (Heidelberg and Berlin) and 3.0-Tesla (London) clinical whole-body CMR-scanners. The referral to DCMR was done according to the available recommendations at the time the study was carried. In this regard, the referring physician evaluated the pretest probability of the patient, and depending on the results of the stress ECG and other factors (i.e. echogenic windows of the patient) the patient was referred for a DCMR examination.

Patients with unstable angina, sub-acute myocardial infarction (within 1 month prior to DCMR), severe arterial hypertension (>200/120 mmHg), moderate or severe valvular disease and general contraindications to CMR (implanted pacemakers or defibrillators, intracranial metal) were excluded.

3104 patients of our cohort have been reported in previous studies [8], [9], [10]. Sixty two additional patients were included in the present analysis.

Traditional risk factors for CAD, including arterial hypertension (blood pressure ≥140/90 mm Hg or antihypertensive therapy) [11], hyperlipidemia (elevated low-density lipoprotein cholesterol (LDL) or statin therapy) [12], current or prior smoking, diabetes mellitus, family history of CAD, prior myocardial infarction and prior revascularization (percutaneous coronary angioplasty (PCI) or coronary artery bypass graft (CABG)) and increased body mass index (BMI>30 kg/m^2^) were recorded at the time of the DCMR. History of CAD encompassed angiographically significant CAD (≥50% lumen narrowing), prior infarction and prior coronary revascularization by PCI or CABG.

### Cardiovascular MR-examination and wall motion analysis

Cardiovascular MR-images were acquired at rest and during a standardized high-dose dobutamine protocol [13]. Vectorcardiographic rhythm and symptoms were monitored continuously, and blood pressure was measured every 3 minutes.

The heart was imaged according to the recommendations of the Society for Cardiovascular Magnetic Resonance using a balanced, fast-field echo sequence with parallel imaging (SENSE, acceleration factor 2) [14]. Typical parameters were a field of view of 400×400 mm^2^, matrix of 256×256 pixels, slice thickness of 8.00 or 10.00 mm, flip angle of 50 degree, time to echo of 1.82 ms, and time to repeat of 3.65 ms. Temporal resolution was 25 to 50 ms. A 4-, 2-, and 3-chamber and 3 short-axis views (apical, mid-ventricular, and basal) were used for wall motion assessment at baseline and were repeated during each stage of stress, including the peak level inotropic stimulation. Dobutamine was infused intravenously during 3-minute stages, at incremental doses of 10, 20, 30, and 40 µg/kg of body weight per minute until at least 85% of the age-predicted heart rate was reached. If at the peak dose of dobutamine infusion the target heart rate was not achieved, atropine was administrated in 0.25 mg increments up to a maximal dose of 2.0 mg. Stress testing was discontinued when the target heart rate was achieved, or when one of the following occurred: new or worsening WMA in at least one myocardial segment, severe chest pain or dyspnea, decrease in systolic blood pressure of ≥40 mmHg, severe arterial hypertension (blood pressure 240/120 mmHg), or severe arrhythmias. In the absence of ischemia, failure to attain 85% of age-predicted maximal heart rate was considered as a non-diagnostic result.

### Image interpretation and analysis

For interpretation of wall motion, corresponding rest and peak stress cine images were displayed using View Forum software (Philips Medical Systems, Best, Netherlands). Seventeen myocardial segments were evaluated at rest and during stress according to AHA guidelines, and wall motion was graded visually using a 4-point scale (0 =  normal wall motion, 1 =  hypokinesia, 2 =  akinesia and 3 =  dyskinesia) [15], [16]. Inducible ischemia was considered present in cases of new or worsening WMA of ≥1 grade during stress in ≥1 segments. Both short and long axis views were considered, and wall motion abnormalities in 1 view were regarded sufficient for the detection of inducible ischemia. For ischemia localization, a 17 segment model was used to localize ischemia according to the distribution of the coronary arteries, with the anterior wall, anteroseptum and LV-apex being ascribed to the left anterior descending artery (LAD), inferoseptum and inferior wall to the right coronary artery (RCA) and inferolateral and anterolateral wall to the left circumflex artery (LCX) [15].

#### Ischemia extent analysis

Patients were categorized in four groups based on the number of segments involved, as follows: (i) no ischemia, (ii) 1 ischemic segment, (iii) 2 ischemic segments and (iv) 3 or more ischemic segments.

#### Ischemia territory analysis

Patients were grouped based on the numbers of coronary territories involved: (i) 1 coronary territory, (ii) 2 coronary territories and (iii) 3 coronary territories.

#### Ischemia localization analysis

This was performed only in patients with one ischemic territory. For this analysis patients were categorized according to inducible ischemia in the LAD versus LCX or RCA perfusion territory.

#### Follow-up data and definition of study endpoints

Personnel unaware of the stress results contacted each subject or an immediate family member (in case of death) and the date of this contact was used for calculating the follow-up time duration. The day of the DCMR examination was considered the start of the follow-up period. Cardiac death and nonfatal myocardial infarction (MI) were registered as hard cardiac events. Cardiac death was defined as death caused by 1) intractable heart failure, 2) acute myocardial infarction, or 3) sudden cause presumably due to infarction or severe arrhythmia. Myocardial infarction was defined by angina of 30 minutes duration and either ST segment elevation of ≥2 mm in 2 consecutive ECG leads or a rise in cardiac enzymes (i.e. troponin T of ≥0.03 µg/l). Other cardiac events included clinically indicated revascularization by PCI or CABG. Hereby, the decision for PCI or CABG was left at the discretion of the referring physician. Because the results of the MR-examination may have triggered coronary revascularization, patients with ‘early’ revascularization within 3 months after DCMR were censored at the time of such revascularization procedures. For patients with an ‘early’ revascularization procedure, a separate analysis was performed to test for the effect of the revascularization procedure on outcomes.

### Statistical analysis

Continuous variables are expressed as mean ± standard deviation, while categorical variables are expressed as median and interquartile range. Unpaired Student *t-*tests or repeated-measures ANOVA with Bonferroni correction for multiple comparisons were used to compare continuous variables. Group differences between ordinal variables were tested using the exact Mann-Whitney test, and differences between nominal variables were assessed using Fisher exact tests. All tests were 2-tailed. In a random subset of cases (n = 150) cine images were re-read by observers blinded to patient identity, clinical and other CMR data (S.K. and G.K.). Agreement between blinded observers and clinical reads for the interpretation of wall motion was calculated using k-statistics. To evaluate the association of the studied parameters with the endpoint, a univariate analysis encompassing the demographic, clinical and CMR-derived parameters was performed. Subsequently, a Cox proportional regression multivariate analysis model with a backward approach with deletion of the least significant variable until all variables had a p<0.1 was generated. Interaction tests were performed to analyze the relationship between presence of ischemia and early revascularization procedures and the extension of ischemia and early revascularization procedures, respectively. Kaplan-Meier curves were used in order to estimate the distribution of cardiac events as a function of the follow-up duration and to calculate the annual rates for cardiac events and revascularization procedures. Statistical analysis was performed using MedCalc 9.3 (MedCalc software, Mariakerke, Belgium) computer program. P-values of <0.05 were considered statistically significant.

## Results

Patients were followed for 3.4±1.8 years (median 3.1; interquartile range 2.0–4.5 years). 187 (5.9%) patients experienced cardiac death and myocardial infarction (82, 2.6% cardiac death and 105, 3.3% nonfatal myocardial infarction). Table 1 summarizes the baseline characteristic of patients with and without cardiac events. 512 (16.2%) (443 by PCI and 69 by CABG) patients underwent early, whereas 258 (9.1%) patients underwent late revascularization procedures. Of patients with known CAD, 1275 (73%) received treatment with ß-blockers, 1254 (72%) with ACE or angiotensin receptor inhibitors, 1289 (74%) with statins and 1351 (78%) with antiplatelet inhibition.

**Table 1:** Baseline characteristic of patients with and without cardiac events.

Parameters	All Patients (n = 3166)	Patients without hard events (n = 2979)	Patients with hard events (n = 187)	*P-values*
	**Clinical data**			
Age	63±12	63±12	66±11	0.006
Male gender; n (%)	2303 (73%)	2153 (72%)	150 (80%)	<0.05
1. Arterial hypertension	2322 (73%)	2171 (73%)	149 (81%)	<0.05
2. Hyperlipidemia	1963 (62%)	1828 (61%)	135 (72%)	<0.01
3. Smoking	950 (30%)	878 (30%)	72 (39%)	<0.05
4. Diabetes mellitus	590 (19%)	534 (18%)	56 (30%)	<0.001
5. Family history	888 (28%)	835 (28%)	53 (28%)	NS
6. Body mass index>30 kg/m^2^	485 (15%)	460 (15%)	25 (13%)	NS
History of CAD	1746 (55%)	1612 (54%)	134 (72%)	<0.001
Prior revascularization	1368 (43%)	1261 (42%)	107 (57%)	<0.001
	**Baseline MR-data**			
LV ejection fraction (%)	58±11.1	58.6±10.8	54.5±13.6	<0.001
LV end-diastolic volume (ml)	159±58	158±58	166±62	<0.01
LV end-systolic volume (ml)	72±55	71±52	83±81	<0.01
	**Cardiac Medications**			
ß-blockers	1936 (61%)	1804 (61%)	132 (71%)	<0.01
ACE inhibitors or angiotensin receptor blockers	2049 (65%)	1925 (65%)	124 (66%)	NS
Statins	1851 (59%)	1722 (58%)	129 (69%)	<0.01
Diuretics	884 (28%)	812 (27%)	72 (39%)	<0.001
Calcium channel blockers	587 (19%)	547 (18%)	40 (21%)	NS
Antiplatelet drugs (aspirin 100 mg or clopidogrel 75 mg)	1980 (63%)	1847 (62%)	133 (71%)	0.01
Nitrates	332 (11%)	299 (10%)	33 (18%)	0.01
	**Baseline Hemodynamics**			
Mean blood pressure (mmHg)	90±16	90±17	88±14	NS
Heart rate (bpm)	67±13	67±13	68±12	NS
Double product (mmHg/min)	8599±2313	8609±2318	8432±2225	NS
	**Stress Hemodynamics**			
Mean blood pressure (mmHg)	110±26	110±23	106±22	0.01
Heart rate (bpm)	131±15	131±15	130±15	NS
Double product (mmHg/min)	19326±4658	19394±4644	18234±4681	<0.01

### Ischemia extension

**Figure 1:**
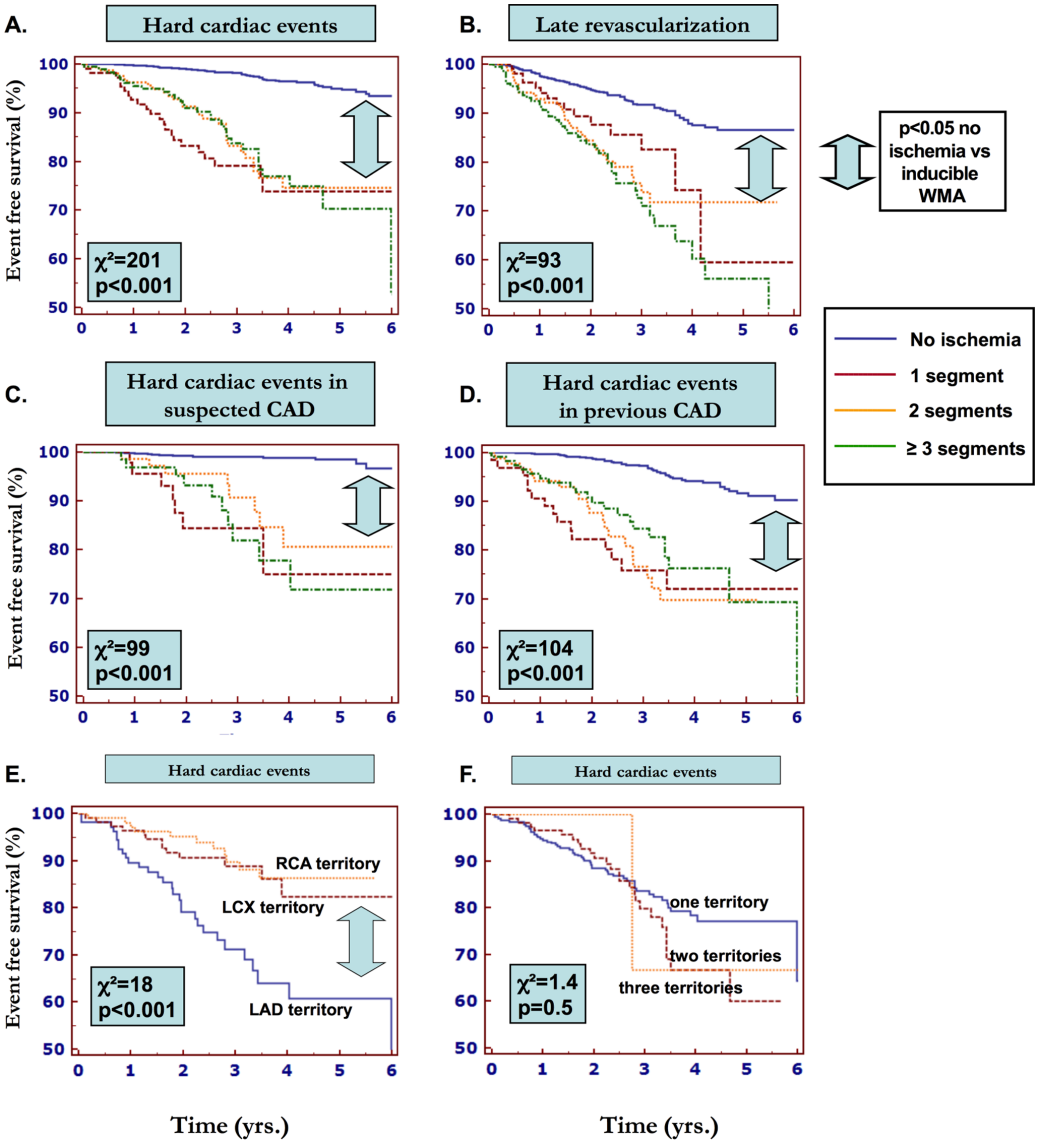
Survival analysis in patients with known or suspected CAD undergoing dobutamine stress CMR. In patients that exhibited dobutamine induced ischemia, the extent of ischemia did not influence hard cardiac events (A) and revascularization procedures (B). The threshold of 1 ischemic segment was enough to predict a poor outcome irrespective of the absence (C) or presence (D) of known CAD. Significantly more hard cardiac events were seen in patients with LAD-territory related ischemia (E). Extension of ischemia to more than one coronary territory did not influence the rate of hard cardiac events (F).

2349 (74.2%) patients had no inducible ischemia. From the 817 (25.8%) patients with inducible WMA, 189 patients (6%) had ischemia in 1 segment, 292 (9.2%) in 2, and 336 (10.6%) in 3 or more segments. Patients with one ischemic segment exhibited a significantly higher yearly rate of hard cardiac events (∼6%), compared to those without inducible ischemia (∼0.6%) but similar to those with ischemia either in 2 (∼5.5%) or at least 3 (∼7%) myocardial segments during DCMR (Fig. 1A). Thus, the event rate within the first 3 years for patients with inducible ischemia, irrespective of extent, was ∼10-fold higher than in patients without ischemia (Table 2). Similar findings were observed for late revascularization (Table 2 and Fig. 1B). Furthermore, one ischemic segment was enough to predict adverse outcomes irrespective of suspected or known CAD (Fig. 1C and 1D, respectively)

**Table 2:** Estimated cumulative cardiac event rates based on extension of inducible WMA.

	Cumulative Event Rates and the corresponding confidence intervals during follow-up (%)					
	1 y	2 y	3 y	4 y	5 y	6 y
	Hard cardiac events					
No inducible WMA (n = 2349)	0.3 (0.1–0.5)	1.0 (0.6–1.4)	1.8 (1.4–2.2)	3.6 (2.6–4.6)	5 (3.6–6.4)	6.6 (4.6–8.5)
WMA in 1 Segment (n = 189)	7.4 (2.5–12.3)	16.8 (9.5–24)	20.9 (12.7–29.1)	26.2 (15.6–36.8)	26.2 (15.6–36.8)	26.2 (15.6–36.8)
WMA in 2 Segments (n = 292)	3.8 (0.86–6.7)	8.7 (4.2–13.2)	16.8 (10.1–23.5)	25.4 (16.4–34.4)	25.4 (16.4–34.4)	25.4 (16.4–34.4)
WMA in 3 or more Segments (n = 336)	4.5 (1.6–7.4)	9.1 (4.9–13.2)	16.3 (10–22.6)	23.1 (14.3–32)	29.7 (17.7–41.6)	29.7 (17.7–41.6)

	Late revascularization procedures					
	1 y	2 y	3 y	4 y	5 y	6 y
No inducible WMA (n = 2349)	2.4 (1.8–2.9)	5.1 (4.1–6)	8.3 (6.9–9.7)	12.5 (9.9–15)	13.5 (10.7–16.2)	13.5 (10.7–16.2)
WMA in 1 Segment (n = 189)	4.8 (0.7–8.9)	12.4 (5.5–19.2)	17.5 (5.9–29)	25.7 (8.2–43.1)	40.6 (11–70.2)	40.6 (11–70.2)
WMA in 2 Segments (n = 292)	7.2 (3.2–11.1)	15.7 (9.4–22)	26.2 (17.4–35)	28.2 (18.8–37.6)	28.2 (18.8–37.6)	28.2 (18.8–37.6)
WMA in 3 or more Segments (n = 336)	8.8 (4.7–12.9)	16.4 (10.7–22)	29 (20.4–37.6)	36.3 (25.1–47.4)	43.8 (29.9–57–7)	62.5 (30.1–94.8)

### Ischemia localization

614 (19.4%) patients showed inducible ischemia in 1, 189 (6%) in 2 and 14 (0.4%) in all 3 coronary territories. Of 614 patients who had inducible ischemia in only one coronary territory, 197 (32%) were related to the LAD, 229 (37%) to the LCX and 188 (31%) to the RCA territory.

Interestingly, the analysis by ischemia localization revealed a higher rate for cardiac death and MI in patients with inducible ischemia within the LAD territory (Fig. 1E). In addition, no difference was noted between patients exhibiting inducible WMA in 1 versus 2 versus all 3 coronary territories (Fig. 1F). Moreover, the proportion of patients who received a late revascularization procedure did not differ in respect to the number of coronary artery territories involved (18% for 1 coronary territory, 23% for 2 coronary territory and 17% for 3 coronary territories, p = 0.4).

### Treatment by early revascularization and its impact on outcomes

From the 512 patients who had an early revascularization procedure, 79 (15.4%) showed ischemia in one territory, 134 (26.1%) ischemia in two territories and 158 (30.8%) in three or more territories. 141 Patients underwent an early revascularization procedure despite negative DCMR.

Neither the extent nor the localization of myocardial ischemia by DCMR influenced the proportion of patients who underwent an early revascularization procedure (p = 0.5 and p = 0.4, respectively).

**Figure 2:**
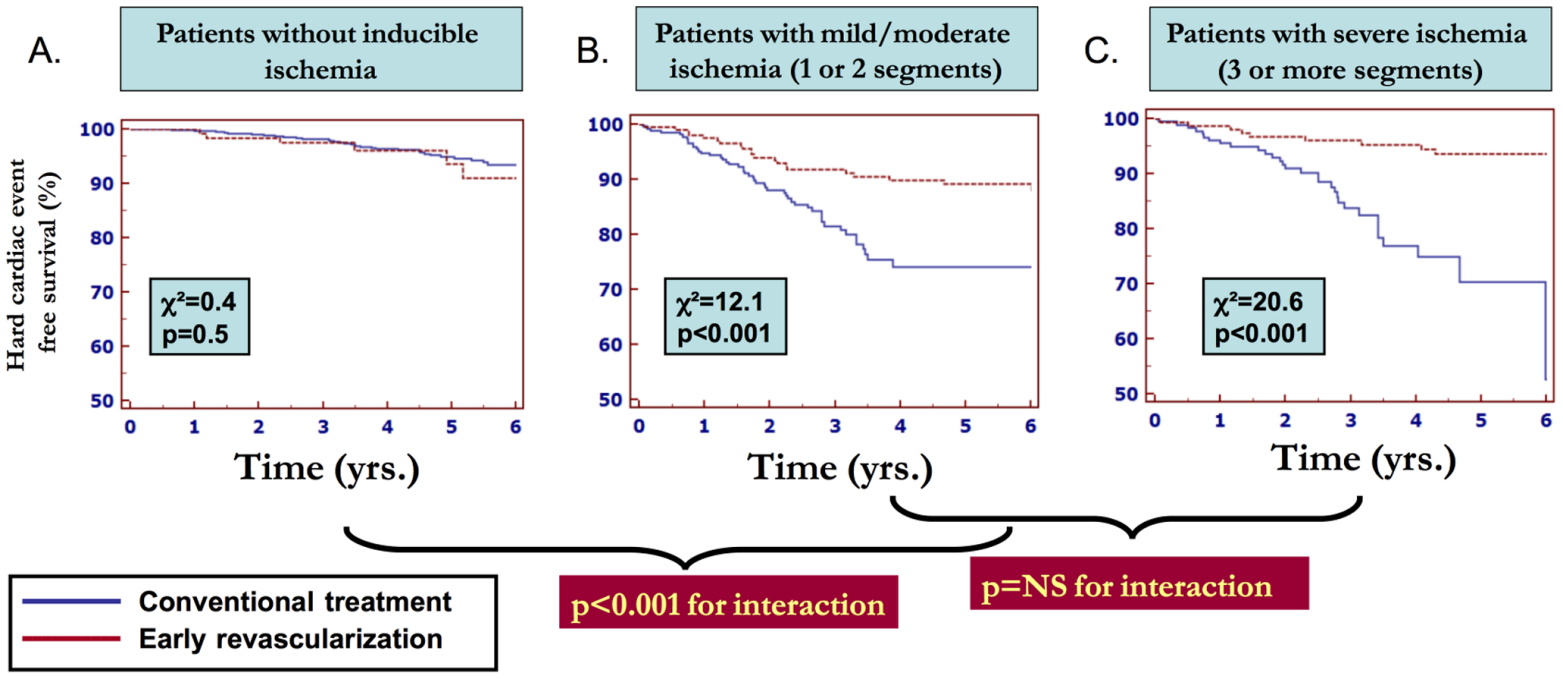
Patients without inducible ischemia do not profit from early revascularization (A). In contrast, patients with either ischemia in 1–2 (B), and ≥3 myocardial segments (C) significantly benefit from early revascularization procedures.

Patients benefited in terms of outcome from early revascularization procedures only if they displayed ischemia on DCMR (Fig. 2A), the benefit being present already with ischemia in 1 or 2 myocardial segments (Fig. 2B, 2C). A test for interaction did not show an influence of the extension of ischemia localization on the benefit of revascularization procedures.

### Univariate and multivariate analysis

As illustrated in Table 3, age, diabetes, smoking, previous CAD, a reduced EF, ischemia in the LAD territory and the presence of inducible WMA were related the predefined endpoints by univariate analysis. By multivariate analysis, inducible WMA was the most robust predictor of hard cardiac events, followed by, a reduced EF, ischemia in LAD territory, the presence of previous CAD, smoking and diabetes mellitus. The presence of more than one segment with ischemia showed no association with the endpoint in both the univariate and multivariate analysis.

**Table 3:** Univariate and multivariate analysis for the prediction of hard cardiac events.

Univariate Analysis			Multivariate Analysis	
Parameter	HR (CI)	p	HR (CI)	p
Age	1 (1–1.03)	0.04		
Gender	1.3 (0.9–2)	0.2		
Diabetes	1.7 (1.2–2.5)	0.003	1.6 (1.1–2.4)	0.008
Smoking	1.6 (1.2–2.3)	0.005	1.6 (1.1–2.4)	0.006
Hyperlipoproteinemie	0.9 (0.6–1.4)	0.7		
Arterial Hypertension	0.9 (0.6–1.4)	0.7		
Family history	1.1 (0.7–1.5)	0.7		
Body mass index>30 kg/m2	0.7 (0.4–1.1)	0.2		
Previous revascularization	1 (0.7–1.5)	0.8		
Previous CAD	1.9 (1.2–3)	0.003	1.7 (1.1–2.5)	0.006
EF rest <50%	2.4 (1.7–3.4)	<0.001	2.4 (1.7–3.5)	<0.001
WMA in more than one segment	0.8 (0.5–1.4)	0.5		
WMA in LAD territory	2 (1.3–3.2)	0.002	2 (1.3–3.2)	0.002
WMA	7.3 (5.1–10.4)	<0.001	5.1 (3.3–7.9)	<0.001

CAD indicates previous coronary artery disease, EF, ejection fraction, LAD left anterior descendent artery and WMA, wall motion abnormalities.

#### Observer variability

Agreement between observers interpreting CMR data in terms of inducible WMA during clinical reads versus blinded reads on a patient level was 94% (weighted k = 0.86; 95%CI = 0.77–0.95).

## Discussion

Our findings in 3166 patients within 3 tertiary centers with high-volume imaging departments demonstrate that:

- (i). The presence of inducible ischemia in only 1 ‘culprit’ myocardial segment during DCMR is sufficient to predict cardiac death and MI in suspected and known CAD.
- (ii). Ischemia within the LAD territory is associated with poorer outcomes.
- (iii). Patients benefit from early revascularization procedures even in the presence of ischemia restricted to 1–2 segments. Conversely, patients without ischemia by DCMR do not benefit from revascularization.

### Ischemia extension and prognosis

The prognostic role of various non-invasive imaging modalities including DSE, nuclear scintigraphy and DCMR in patients with CAD is clinically established [8], [17], [18], [19]. According to current guidelines, the presence of 10% ischemic myocardium is translated to ≥2 myocardial segments with inducible perfusion deficits or of ≥3 segments with inducible wall motion abnormalities with other imaging modalities like DSE, DCMR and vasodilator stress perfusion CMR [7]. However, from a pathophysiologic point of view, inducible WMA occur later in the ischemic cascade than perfusion defects, thus being a less sensitive, albeit highly specific for myocardial ischemia by CMR [4], [20]. Therefore, one myocardial segment with inducible WMA may correspond to more than one segments with perfusion defects by vasodilator stress CMR or to a ≥10% myocardium by nuclear imaging modalities [21]. In this regard, very few studies addressed the question whether the extent and localization of ischemia influence clinical outcomes so far. Using DSE, Marwick et al showed a worse prognosis for patients with inducible ischemia in more than one coronary territory [6]. In the same line, Hachamovitch et al showed that the extent of ischemia is related to the occurrence of hard cardiac events using SPECT [19]. In a previous CMR study however, the number of ischemic segments in terms of WMA during DCMR was not associated with cardiac outcomes [22]. In a more recent CMR study on the other hand, ischemia during vasodilator stress in ≥1.5 myocardial segments was found to be predictive of poor outcomes irrespective of CAD presence or absence [23].

In our study we demonstrated in a large cohort of over 3000 patients, that even a single segment of the myocardial circumference exhibiting ischemia during DCMR translates in a much higher rate of cardiac death and MI. The presence of ischemia in two or more segments however, did not further enhance the associated risk for future events, compared to patients with ischemia in a single myocardial segment. DCMR was significantly associated with outcomes both in patients with suspected and known CAD. Importantly, in contrast to previous nuclear and echocardiography studies an association between ischemic burden and outcomes could not be established, as any evidence of ischemia was predictive of markedly enhanced risk. On the other hand, myocardial perfusion during DCMR was not systematically analysed in our study, which is a limitation. However, the assessment of myocardial perfusion is still challenging with increasing heart rates during dobutamine due to motion artefacts. In addition, with current standard perfusion protocols, less myocardium can be visualized, so that ischemia in regions like the apical cap or the true basal inferior wall may be missed. These shortcomings, however, may be circumvented by the recent availability of multichannel cardiac coils, which may allow for 3D first-pass perfusion scans.

Furthermore, a recent comparison of DSE and DCMR showed the latter to be a more robust predictor of adverse outcome, which could be explained by the better spatial resolution of CMR resulting to a lower likelihood for false positive results compared to DSE [24].

### Ischemia localization and prognosis

Analysing by ischemia localization we found a higher likelihood of cardiac events in patients with inducible WMA in the left anterior descending territory. Our findings are in agreement with previous reports, where a higher rate of adverse cardiac events was noticed in patients with angiographically significant LAD stenosis compared to significant lumen narrowing in other coronary vessels [25], [26]. Moreover, a trend for poorer outcomes in patients with LAD-related ischemia was also previously elegantly shown in a DCMR study [22]. The impact of localization on prognosis may be attributed to a higher risk for developing larger transmural MI areas with consecutive poor ejection fraction and congestive heart failure in patients with LAD related ischemia [27], [28].

### Revascularization procedures and prognosis

In our study, early revascularization procedures reduced cardiac event rates in patients with inducible ischemia in ≥1 myocardial segments, which is in agreement with recent CMR trials [23] and the FAME 2 trial which highlighted the beneficial effect of revascularization procedures only in patients with positive FFR [29]. In a recent subsection analysis of the ‘COURAGE’ trial on the other hand, Shaw et al reported that neither the presence nor the extent of ischemia predicts the likelihood of future cardiac events [30]. Of course it needs to be considered that in contrast to Shaw et al, our study had an observational character and DCMR results were not used in order to structure patient treatment in a blinded or randomised way. Interestingly, with our cohort the beneficial effect of revascularization procedures was present already in patients with ‘mild’ ischemia in only 1 or 2 segments, which also confirms the fact that ischemia by WMA is decisive for future events even if observed in a single myocardial segment.

### Limitations

Our study had an observational character, and DCMR results were not used in order to structure patient treatment in a blinded or randomised way. In this regard, clinicians had full access to the results of stress testing, which obviously triggered early revascularization procedures in a large percentage of patients with inducible ischemia. However, subsection analysis showed that neither the extent nor the localization of ischemia influenced the referral of patients to an early revascularization procedure. Although patients who underwent such early revascularization procedures were censored from analysis at this time point we cannot exclude some influence to our results due to referral or selection biases. Moreover patients were not randomly assigned to undergo invasive versus medical therapy, which may have influenced the obtained results due to selection biases.

## Conclusions

In our observational study, one single myocardial segment with inducible wall motion abnormality during DCMR is sufficient to predict hard cardiac events and revascularization procedures in patients with known or suspected coronary artery disease. Furthermore, LAD territory related ischemia is associated with a worse prognosis compared to the other coronary territories. Patients with inducible ischemia benefit from coronary revascularization even in case of ‘mild extent’ ischemia in 1–2 myocardial segments, whereas those without ischemia do not benefit from coronary revascularization and should therefore be treated conservatively.
